# CRISPR-Cas systems feature and targeting phages diversity in *Lacticaseibacillus rhamnosus* strains

**DOI:** 10.3389/fmicb.2023.1281307

**Published:** 2023-12-06

**Authors:** Bahman Panahi, Behnaz Dehganzad, Yousef Nami

**Affiliations:** ^1^Department of Genomics, Branch for Northwest and West Region, Agricultural Biotechnology Research Institute of Iran (ABRII), Agricultural Research, Education and Extension Organization (AREEO), Tabriz, Iran; ^2^Department of Natural Sciences, University of Tabriz, Tabriz, Iran; ^3^Department of Food Biotechnology, Branch for Northwest and West Region, Agricultural Biotechnology Research Institute of Iran (ABRII), Agricultural Research, Education and Extension Organization (AREEO), Tabriz, Iran

**Keywords:** CRISPR, genome, immune system, phage, spacer

## Abstract

One of the most important adaptive immune systems in bacteria against phages is clustered regularly interspaced short palindromic repeats (CRISPR) and CRISPR-associated (CAS) genes. In this investigation, an approach based on genome mining was employed to characterize the CRISPR-Cas systems of *Lacticaseibacillus rhamnosus* strains. The analysis involved retrieving complete genome sequences of *L. rhamnosus* strains, and assessing the diversity, prevalence, and evolution of their CRISPR-Cas systems. Following this, an analysis of homology in spacer sequences from identified CRISPR arrays was carried out to investigate and characterize the range of target phages. The findings revealed that 106 strains possessed valid CRISPR-Cas structures (comprising CRISPR loci and Cas genes), constituting 45% of the examined *L. rhamnosus* strains. The diversity observed in the CRISPR-Cas systems indicated that all identified systems belonged to subtype II-A. Analyzing the homology of spacer sequences with phage and prophage genomes discovered that strains possessing only CRISPR-Cas subtype II targeted a broader spectrum of foreign phages. In summary, this study suggests that while there is not significant diversity among the CRISPR-Cas systems identified in *L. rhamnosus* strains, there exists notable variation in subtype II-A systems between *L. rhamnosus* and other lactobacilli. The diverse nature of these CRISPR-Cas systems underscores their natural activity and importance in adaptive immunity.

## Introduction

The widespread occurrence of malignant bacteriophages is one of the most serious issues we confront in food fermentation. This might affect the quality of fermented products or cause manufacturing operations to be delayed. Although preventative methods to manage bacteriophages have been implemented since their identification as the primary cause of fermentation failure, bacteriophages continue to represent a significant concern to the dairy industry (Forde and Fitzgerald, 1999). The lactic acid bacteria (LAB) industry faces a severe problem with phage contamination (Garneau and Moineau, 2011; Kiani et al., 2021). Phages have the ability to lyse bacteria and affect their death, reduce the number of living bacteria, slow down the fermentation process, and even cause production failure. As a result, these disadvantages lead to a decrease in acid production and taste. Because phages may withstand pasteurization, they are difficult to eradicate entirely. They are able to expand rapidly and even destroy the entire production chain, which causes great economic losses; therefore, the anti-phage ability of LAB needs to be investigated to solve the problem they are facing and the useful applications they can have. Since the fermentation conditions are optimized to increase the growth of starter cultures, however, the presence of a large number of actively growing bacterial isolates creates a suitable substrate for the multiplication of bacteriophages. Bacteriophages may cause incompatibility or fermentation failure and reside in a given plant for several years (Lavelle et al., 2018; Nami et al., 2021).

Bacteria have developed several mechanisms to escape the attack of bacteriophages. Phage resistance mechanisms exhibit a wide range of diversity and may be chosen to specifically target certain bacteriophages or groups. Many of these mechanisms have been identified on plasmids, especially for lactococci (Mills et al., 2011), while other resistance mechanisms exist on bacterial chromosomes. These phage resistance mechanisms can be categorized into inhibition of bacteriophage uptake, inhibition of bacteriophage genome injection, restriction modification systems with incomplete infectivity, and short palindromic repeat systems (CRISPR/Cas) (Garneau et al., 2010).

In the context of *L. rhamnosus*, a probiotic bacterium commonly found in the human gut, CRISPR-Cas systems play essential roles including immune defense, maintenance of genome integrity, probiotic engineering, functional genomics, study of host–microbe interactions, enhanced probiotic strains, biotechnology and bioprocessing, and studying microbiome dynamics (Guo et al., 2023). CRISPR-Cas systems play significant roles in the context of *L. rhamnosus* by both protecting the bacterium from invading DNA and allowing for applications in microbiology, biotechnology, and the study of host–microbe interactions, making them invaluable tools for understanding and utilizing probiotic bacteria like *L. rhamnosus*.

Yoshizumi Ishino, a Japanese scientist, discovered the CRISPR-Cas system in 1987 after finding comparable DNA sequences in the genome of the *Escherichia coli* bacterium while looking for genes involved in phosphate metabolism. These bacterial genomic sequences were dubbed CRISPR. The actual function of this unique system remained unclear until the mid-2000s, and later, these sequences were identified in the genomes of other bacteria, including halophilic archaea. These sequences are crucial to a living organism’s evolutionary connection (Khan et al., 2022). The CRISPR system consists of clustered short palindromic repeats with regular spacing and CRISPR-related genes called Cas proteins, which form an adaptive defense system in bacteria and archaea. CRISPR systems are unique among bacteriophage resistance systems in that they can continuously update and adjust their immune responses to match the nucleic acid sequence of invaders. This system consists of repetitive parts and spacer sequences in a CRISPR locus (Chyou and Brown, 2019). CRISPR repeats are short conserved sequences of 20–40 bases. These repeats separated by unique sequences called spacers. The spacer sequences are obtained often from an invasive plasmid or bacteriophage and are incorporated into the bacterial crisper (Kahraman Ilıkkan, 2022).

The CRISPR system functions through three distinct phases: adaptation, processing, and interference. The adaptation module, responsible for spacer insertion, consists of Cas4, Cas1, and Cas2. Cas1, operating as an integrase during adaptation, is a metal-dependent deoxyribonuclease (Hudaiberdiev et al., 2017). Cas2 tightly associates with Cas1 to form a cohesive complex, whereas Cas4 functions as a nuclease, playing a role in the cleavage of single-stranded DNA (ssDNA). Moreover, Cas4 is involved in the processing of protospacers and the identification of PAM sequences, as outlined by Behler et al. (2018). On the other hand, the Cas9 protein forms the essential component of the processing module, playing a critical role in pre-crRNA processing. The module responsible for recognizing and cleaving targets comprises Cas11, Cas10, Cas8, Cas5, and Cas3 in class I, while class II involves Cas13, Cas12, or Cas9. Cas3, serving as both a nuclease and helicase, plays a crucial role in the interference process. In subtype I-C, it has been noted that Cas5m assumes the role typically carried out by Cas6. Additionally, within the interference module, Cas8 participates in the recognition of PAM sequences (Makarova et al., 2020).

The CRISPR system includes two classes [class I and II (6 types)] and 34 subgroups. In the class I system, there are multi-subunit complexes of Cas proteins, but in class II, a single Cas protein, Cas9, performs all of the effector complex’s actions. Types I, III, and IV comprise Class I, whereas Types II and V comprise Class II. Each of these five types has its own pattern of expression, interaction, and adaptability. In short, the presence of Cas3 genes indicates class I, Cas9 indicates class II, Cas10 indicates class III, and types IV and V are hypothetical groups and lack functional characteristics (Makarova et al., 2018).

LAB are significant bacteria that primarily create lactic acid as a byproduct of their metabolic activity and they serve a diverse and vital function in agriculture, the food industry, and medicine. LAB usually provide the energy they need by fermenting sugars, as a result of which they produce lactic acid. These bacteria participate in the fermentation of many foods, and it can be said that fermentation using them is one of the most common and well-known tasks in food preservation. The food industry is always looking for isolates with better traits and qualities to improve product quality. Furthermore, these bacteria possess medicinal qualities that are critical for improving human health (Bintsis, 2018). Although there are over 60 genera in this group, the *Lactobacillus*, *Pediococcus*, *Streptococcus*, *Lactococcus*, *Enterococcus*, and *Leuconostoc* genera are usually involved in food fermentation (George et al., 2018).

Probiotics are living microorganisms that, when taken, may have health advantages. Such characteristics give *L. rhamnosus* a better chance of survival and thus may provide long-term benefits (Corcoran et al., 2005). One of the well-studied probiotic bacteria is *L. rhamnosus*, which is added to various foods as a food supplement (Yan and Polk, 2012). *L. rhamnosus* is available as a probiotic supplement and is often added to yogurt, cheese, milk, and other dairy products to increase their probiotic content. It can also be added to dairy products for other reasons. For example, this bacterium, which is a non-initiator lactic acid bacterium, plays a key role in cheese ripening, which increases the flavor, and in late-ripening cheeses, due to its ability to adapt to changing environmental conditions. However, many products containing *L. rhamnosus* usually do not list it in the ingredient list (Liptáková et al., 2008; Lazzi et al., 2014). *L. rhamnosus* is a LAB that is found in many types of environmental habitats, such as dairies, crafts, and industry, the oral cavity, the intestinal tract, and the vagina (Douillard et al., 2013). This bacterium, which belongs to the genus *Lactobacillus*, produces the lactase enzyme, which breaks down lactose sugar, which is found in dairy products, into lactic acid. Bacteria of this genus, such as *L. rhamnosus*, are considered probiotics.

As far as we are aware, limited data exists regarding the variety and progression of the CRISPR-Cas system in *L. rhamnosus*. Consequently, in this present investigation, we employed an extensive genome mining approach to delineate the prevalence, diversity, and various features of CRISPR-Cas systems, along with their inherent defense mechanisms against phages.

## Materials and methods

### Eligible data retrieval

In order to collect the genomic sequences of different strains of *L. rhamnosus* for the analysis of this research, complete genome sequences along with their annotations and other related information such as the percentage of GC and other features of the genomes were collected from the NCBI database and used in the current study.

### Prediction of CRISPR/Cas systems

Initially, MetaCRT (Moller and Liang, 2017) was used to detect minor repeats and predict CRISPR arrays in bacterial genomes. Due to repetitions with excessive mutations or long spacers, a long CRISPR may be divided into multiples in certain circumstances. CRISPRs that are close to one another and sequences are used to prevent such situations. A single CRISPR was defined as a highly comparable repeat that shared the same locus. In the next step, conserved repeats were determined for each CRISPR array and clustered using CD-HIT-EST (Li and Godzik, 2006). Then hmmscan (Finn et al., 2011) was used to search for Cas proteins related to the identified systems, and they were attributed to one of the universal protein categories: Cas1, Cas5, Cas7, Cas8, Cas9, Cas10, Csf1, and Cpf1.

### Determining the type of CRISPR/Cas

A CRISPR-Cas locus consists of the CRISPR locus and the Cas genes nearby. The CRISPRone server (Zhang and Ye, 2017) was used to determine the type of this system. The Cas gene determines the type of CRISPR/Cas locus at each location. Based on the Cas gene sequence similarity, subtype designation was performed using the blast program. The predicted CRISPR locus in the collected genomes was further validated using CRISPR-Cas^++^.^1^

### Analysis of protospacers and adjacent motifs

The verification of protospacer identity from phage genomes involved the utilization of BLASTn, employing the settings blastn -evalue 1e-3 -remote -db nt -outfmt 5 as specified in CRISPRutils. Protospacers exhibiting over 85% identity and fewer than 3 mismatches were carefully considered for subsequent analysis. In the continuation of the analysis of this stage, heat map software implemented in R packages (Villanueva and Chen, 2019) was used to categorize and cluster the identified targets and to convert the number and type of identified targets quantitatively. To determine the protospacer adjacent motif (PAM), the 5′ and 3′ ends of protospacer sequences were aligned with a 10-nucleotide flank on each side. The WebLogo was employed to visually represent the identified PAM sequence.

### Prophages identification

In order to quickly find prophage sequences in bacterial genomes, the GLIMMER software (Kelley et al., 2012) was used to show potential prophages based on their typical protein structures. In the next step, the BLASTP software (Johnson et al., 2008) based on the genome database of the identified viruses was used to determine and introduce the family and type of the identified prophages.

### Phylogeny analysis

In order to understand the evolutionary relationships of the anti-phage systems identified in the studied isolates, classification methods based on the genetic distance between associated protein amino acid sequences were used. First, the multiple sequence alignments were done using CLUSTALW software, and then the genetic distance was calculated using the neighbor joining algorithm. Then, a phylogeny tree was constructed using Mega 7.0 software.

## Results and discussion

### Presence and variety of CRISPR-Cas systems within *Lacticaseibacillus rhamnosus*

The examination involved the analysis of 238 *L. rhamnosus* strains’ genomes to ascertain the presence, prevalence, and attributes of CRISPR-Cas systems. Results indicated that 106 strains harbored authentic CRISPR-Cas structures, encompassing both CRISPR loci and Cas genes, constituting 45 percent of the scrutinized *L. rhamnosus* strains. The observed frequency of CRISPR loci aligned with the average frequency documented for other bacteria, standing at 45% (Grissa et al., 2007). The presence or absence of CRISPR-Cas systems in different strains of *L. rhamnosus* can be due to a variety of factors, including the evolutionary history of the strains, environmental pressures, and genetic variation (Nami et al., 2023). The implications of such variability can be enlightening in phage defense, genetic diversity and transferred horizontally between bacterial strains. Additionally, understanding this variability can provide insights into the complex dynamics of the human microbiome (Gilbert et al., 2018).

Strains with the CRISPR array underwent further assessment to confirm the presence of Cas genes and determine subtype designation, utilizing algorithms incorporated in the CRISPR-Cas Finder and CRISPRone server applications. Among the 106 *L. rhamnosus* strains displaying a complete CRISPR-Cas system, as detailed in Table 1 and Supplementary Table S1, all strains were classified under subtype II-A. As depicted in Supplementary Figure S1, subtype II-A encompasses Cas2, Cas1, Cas9, and the characteristic Cas gene (Csn2). Consistent with earlier research, the outcomes suggested the prevalence of subtype II-A in LAB (Yang et al., 2020; Kahraman Ilıkkan, 2022; Panahi et al., 2022). Previous studies have focused on identifying and characterizing CRISPR-Cas systems in diverse bacteria (Van Der Oost et al., 2014; Hidalgo-Cantabrana et al., 2019). Our analysis indicated that *L. rhamnosus* exclusively harbors subtype II-A in its CRISPR-Cas system, contrary to prior findings that subtype I-E is the most commonly observed subtype among *Lactobacillus* strains (Karvelis et al., 2013).

**Table 1:** CRISPR subtypes, direction and corresponding *L. rhamnosus* strains.

Strains	CRISPR type	Direction	Strains	CRISPR type	Direction
HN001	Subtype-II-A	+	IBL027	Subtype-II-A	−
GG (ATCC 53103)	Subtype-II-A	−	LR5	Subtype-II-A	−
ATCC 53103	Subtype-II-A	−	DSM 14870	Subtype-II-A	−
R0011	Subtype-II-A	−	B1	Subtype-II-A	+
ATCC 21052	Subtype-II-A	−	P5	Subtype-II-A	−
LOCK900	Subtype-II-A	−	P4	Subtype-II-A	−
PEL5	Subtype-II-A	−	P1	Subtype-II-A	−
PEL6	Subtype-II-A	−	P3	Subtype-II-A	−
K32	Subtype-II-A	+	UMB0004	Subtype-II-A	+
116	Subtype-II-A	−	LR2	Subtype-II-A	−
BPL15	Subtype-II-A	+	DS12_11	Subtype-II-A	−
313	Subtype-II-A	−	DS9_11	Subtype-II-A	−
Lr138	Subtype-II-A	−	DS18_11	Subtype-II-A	−
319_LRHA	Subtype-II-A	−	DS3_11	Subtype-II-A	+
186_LRHA	Subtype-II-A	−	DS4_11	Subtype-II-A	−
784_LRHA	Subtype-II-A	−	GG	Subtype-II-A	−
893_LRHA	Subtype-II-A	−	ARJD	Subtype-II-A	+
944_LRHA	Subtype-II-A	+	LR-B1	Subtype-II-A	−
979_LRHA	Subtype-II-A	−	LR-CVC	Subtype-II-A	−
214_LRHA	Subtype-II-A	+	LR-B2	Subtype-II-A	+
390_LRHA	Subtype-II-A	+	LR-GG-MoProbi	Subtype-II-A	−
389_LRHA	Subtype-II-A	−	LR-S	Subtype-II-A	−
BPL5	Subtype-II-A	+	1.0320	Subtype-II-A	−
Lrh11	Subtype-II-A	−	AMBR1	Subtype-II-A	−
Lrh22	Subtype-II-A	−	AMBR5	Subtype-II-A	−
Lrh7	Subtype-II-A	−	AMBR6	Subtype-II-A	+
Lrh14	Subtype-II-A	+	AMBR7	Subtype-II-A	+
Lrh28	Subtype-II-A	+	MGYG-HGUT-01293	Subtype-II-A	−
Lrh18	Subtype-II-A	−	hsryfm 1,301	Subtype-II-A	+
Lrh32	Subtype-II-A	+	BIO6870	Subtype-II-A	−
Lrh8	Subtype-II-A	−	IDCC 3201	Subtype-II-A	−
Lrh19	Subtype-II-A	+	LV108	Subtype-II-A	−
Lrh17	Subtype-II-A	+	OSU-PECh-69	Subtype-II-A	+
Lrh9	Subtype-II-A	+	DPC 7102	Subtype-II-A	+
Lrh34	Subtype-II-A	−	CBC-LR1	Subtype-II-A	+
Lrh16	Subtype-II-A	−	JL-1	Subtype-II-A	−
Lrh6	subtype-II-A	−	TK-F8B	Subtype-II-A	−
Lrh4	Subtype-II-A	−	1001095A_150126_D4	Subtype-II-A	−
Lrh3	Subtype-IV-A	+	1001287B_170213_A1	Subtype-II-A	+
Lrh1	Subtype-II-A	−	1001216B_150713_B1	Subtype-II-A	−
LRB	Subtype-II-A	−	1001270B_150601_F12	Subtype-II-A	+
HCT70	Subtype-II-A	+	B6	Subtype-II-A	−
ASCC 3029	Subtype-II-A	−	ERR1430462-bin.4	Subtype-II-A	−
RI-004	Subtype-II-A	−	1,473	Subtype-II-A	−
AMC143	Subtype-II-A	−	LDTM7511	Subtype-II-A	+
BFE5264	Subtype-II-A	−	CE1	Subtype-II-A	−
L156.4	Subtype-II-A	−	AS	Subtype-II-A	−
WQ2	Subtype-II-A	−	L3_114_000M1_dasL3	Subtype-II-A	+
Pen	Subtype-II-A	+	L3_079_062G2_dasL3	Subtype-II-A	−
Lrh46	Subtype-II-A	+	Lr-G14	Subtype-II-A	+
Lrh38	Subtype-II-A	+	RAB2019A	Subtype-II-A	+
Lrh39	Subtype-II-A	−	F	Subtype-II-A	−
4B15	Subtype-II-A	+	ICIS-627	Subtype-II-A	−

**Figure 1:**
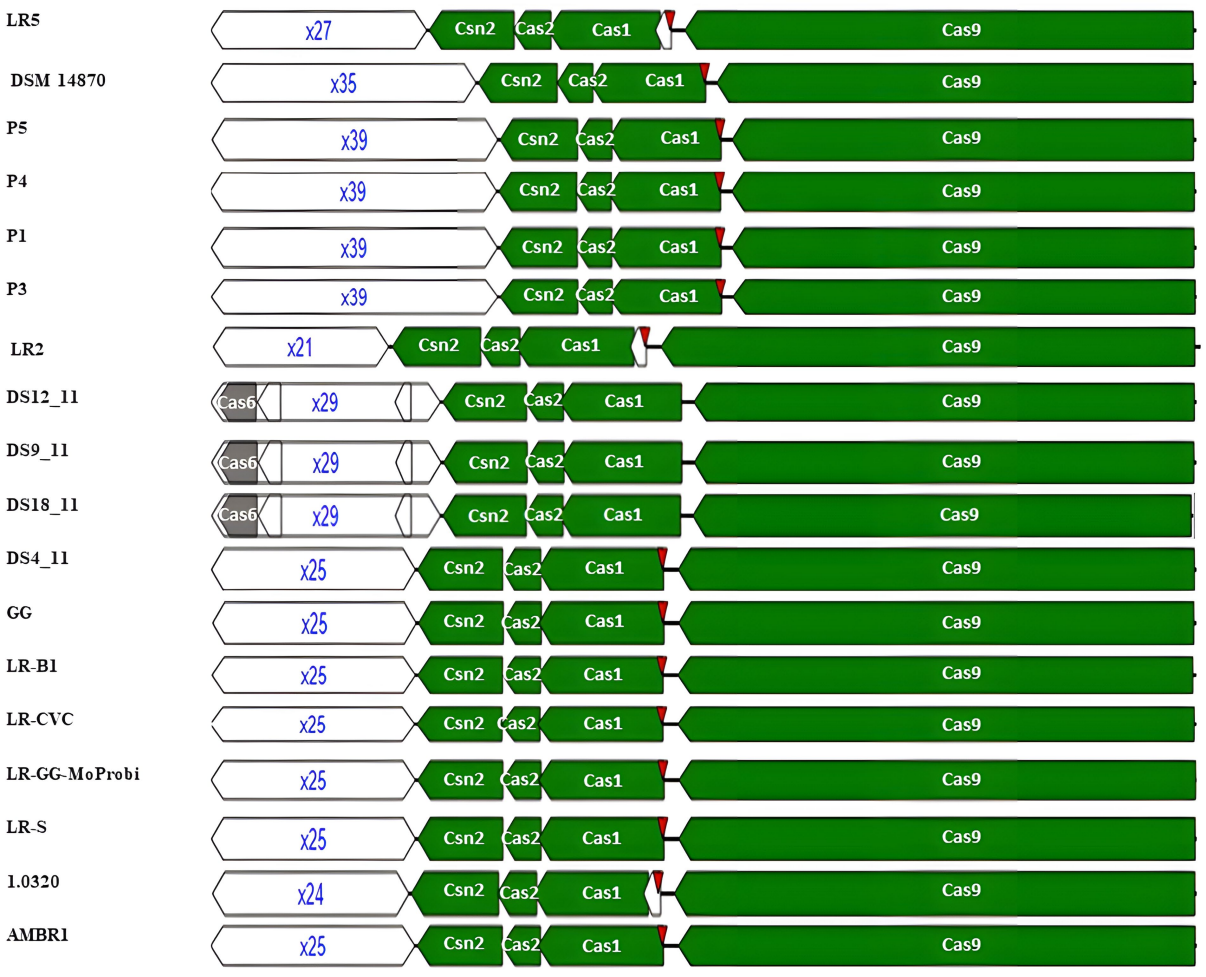
Structural variation of identified CRISPR-Cas subtype II-A in *L. rhamnosus.*

The execution of adaptation, expression, interference, signal transduction, and ancillary functions was carried out using the CRISPR-associated proteins known as Cas proteins (Tang et al., 2017). Examination of the encoded Cas genes within identified CRISPR-Cas systems revealed distinct features in each subtype’s functional modules. The “core” adaptation module, requiring spacer insertion, is composed of Cas1 and Cas2 (Lee et al., 2019). Cas1, functioning as a deoxyribonuclease, acts as an integrase during adaptation (Marraffini and Sontheimer, 2010). According to the genome mining approach, the adaptation module in *L. rhamnosus* CRISPR-Cas systems of subtype II-A includes an integrase (Cas1) and a structural component (Cas2). Interestingly, all 106 strains possessed Cas1 and Cas2, with the exception of strain 389_LRHA, which lacked Cas2. The Cas9 protein, constituting the processing module, contributes to pre-crRNA processing (Behler et al., 2018). Our results revealed that, excluding strain AMBR6, all strains carrying subtype II-A included the Cas9 protein in their CRISPR systems. The interference module aids in target identification and cleavage, with Cas9, Cas12, or Cas13 for class II (Makarova et al., 2020). Cas12 and Cas13, classified under class II, have distinct roles in the CRISPR system. Cas12 is involved in cleaving both strands of pre-crRNA, while Cas13 is responsible for processing pre-crRNA, as discussed by O’Connell (2019). While Cas9 was a prevalent component of the interference module in all studied strains, Cas12 and Cas13 were only found in the AMBR6 strain (Figure 1).

The diversity of various modules in CRISPR-Cas systems plays a crucial role in the context of adaptive immunity. It was demonstrated that the diversity of these capture complexes allows the system to recognize and adapt to a wide range of invasive elements. Moreover, the diversity of effector modules ensures that a variety of invaders can be effectively neutralized. Structure variation of CRISPR arrays leading to acquiring new and diverse spacers (sequences from invaders) differ between systems. This diversity allows for efficient adaptation to a continually evolving set of invaders (Van Der Oost et al., 2014; Hidalgo-Cantabrana et al., 2019).

### Protospacer adjacent motif analysis

**Figure 2:**
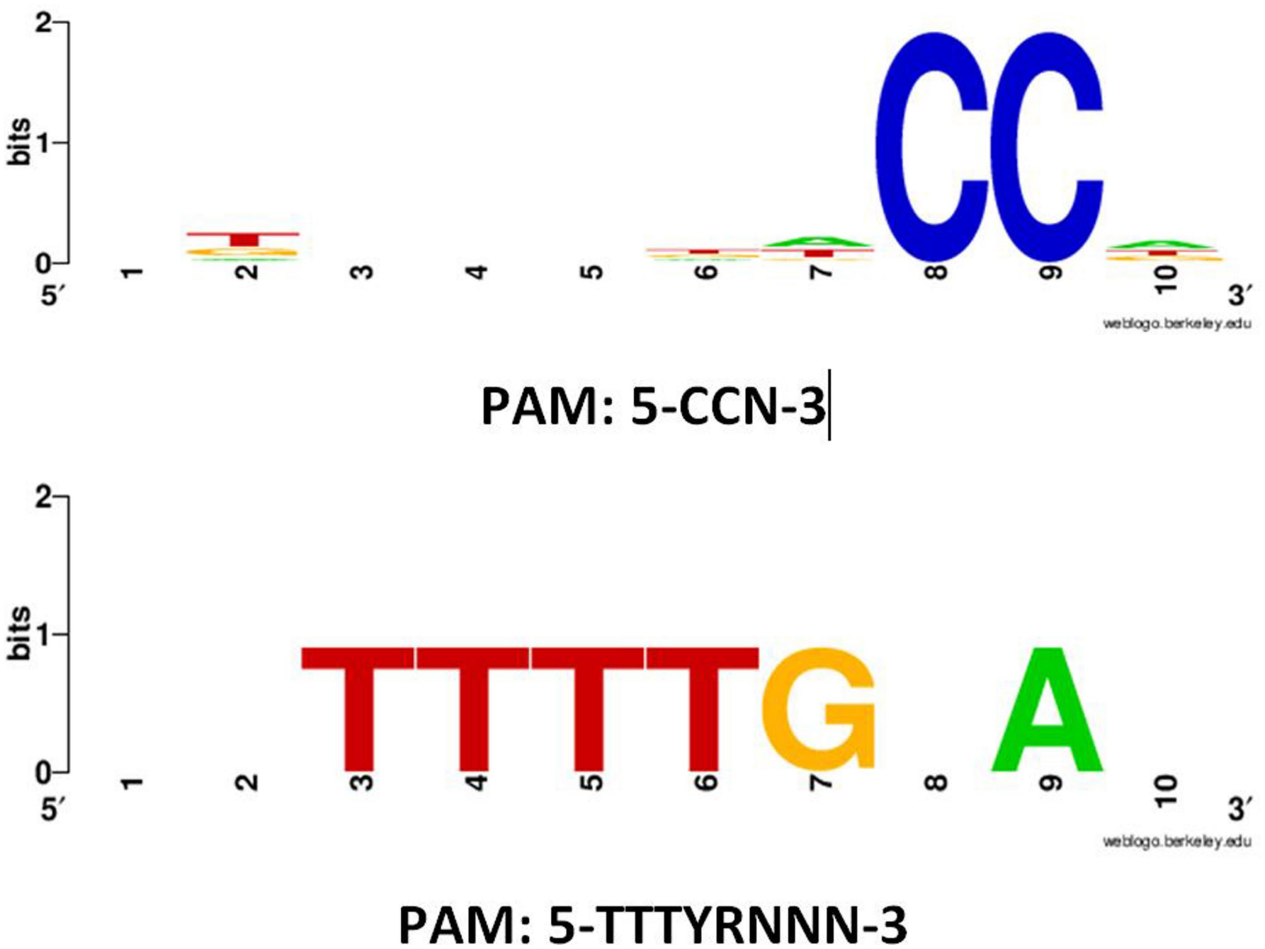
Predicted PAM motifs in 5′ flanking regions of protospacers in subtypes II-A CRISPR-Cas systems in *L. rhamnosus* strains. The height of each nucleotide indicated the frequency of nucleotide occurrence.

PAM motifs are essential for the functionality and specificity of CRISPR-Cas systems. They ensure precise target recognition, prevent self-targeting, and provide the basis for controlled and specific genome editing. Understanding and manipulating PAM sequences are central to harnessing the full potential of CRISPR-Cas technology for various applications in genetic engineering, biotechnology, and medicine (Mojica et al., 2009). Detected PAM motifs in 5′ flanking regions of protospacers in subtype II-A CRISPR-Cas systems are presented in Figure 2, respectively. For subtype II-A, two types of PAM motifs, including 5′-CCN-3′ and 5′-TTTYRNNN-3′, were detected in the 5′ flanking regions of protospacers. For 5′-CCN-3′ and 5′-TTTYRNNN-3′ motifs, about 1,165 and 3 sequences, respectively, were found. The height of the nucleotides in the web logo output indicated the frequency of occurrence of each nucleotide. In previous studies, it has been reported that the 5′-NGG-3′ is the most frequent PAM motif in the CRISPR-Cas subtype II-A in other *Lactobacillus* species such as *Levilactobacillus brevis* and *Lacticaseibacillus casei* (Yang et al., 2020; Panahi et al., 2022). The occurrence analysis of each PFAM motifs showed that about 500 and 3 CRISPR-Cas contain 5′-CCN-3′ and 5′-TTTYRNNN-3′ motifs, respectively, which highlighted the dominance of the 5′-CCN-3′ motifs in *L. rhamnosus* strains CRISPR-Cas systems. The absence of this motif was noted in the 5′ flanking regions of protospacers within the CRISPR-Cas systems subtype II-A of *L. rhamnosus* strains. Contrary to our observations, previous reports indicated the presence of 5′-CCN-3′ and 5′-CC-3′ motifs in *L. brevis* CRISPR-Cas subtype II-A subtypes (Panahi et al., 2022). In line with our findings, 5′-TTTYRNNN-3′ has been reported for the 5′ flanking regions of protospacers in *L. brevis* strain CRISPR-Cas systems (Panahi et al., 2022). The presence or absence of specific PAM motifs in the CRISPR system of bacterial species, such as *L. rhamnosus*, can be attributed to accumulate mutations and variations in CRISPR systems, phage-host coevolution, horizontal gene transfer, where genetic material is exchanged between different organisms (Makarova et al., 2020). Furthermore, the presence or absence of certain PAM motifs may be functionally advantageous for the specific needs of *L. rhamnosus*. Different PAM motifs may have different recognition specificities, and their presence may enhance the bacterial defense system against particular phages or genetic elements (Panahi et al., 2022; Nami et al., 2023). In this case, the evolution of these motifs would be driven by their utility in defending against specific threats. Some bacterial species may have genomic constraints that limit their ability to accommodate certain PAM motifs. Moreover, the size and structure of the CRISPR locus, as well as the available genetic material, can influence which PAM motifs can be incorporated or maintained in a genome (Nami et al., 2023).

### Potential targeting phages and prophages

**Figure 3:**
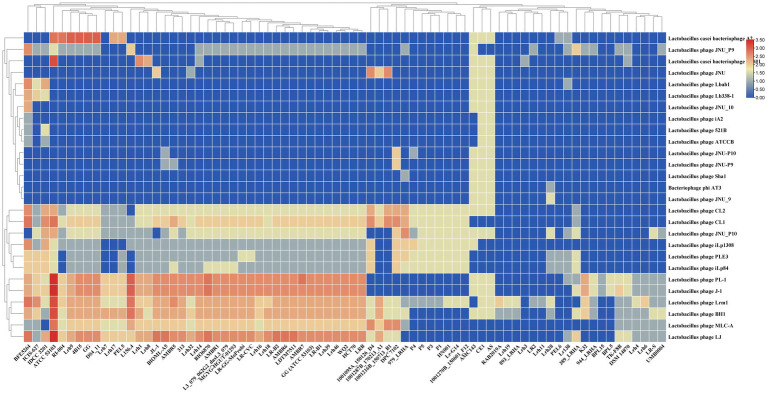
Diversity of potential phages and prophages targeting by CRISPR-Cas systems subtype II-A in *L. rhamnosus* strains. Rows and columns of the figure represent the targeting phages and studied strains name. The cells color represents the targeting number occurrence between spacers and phages. The number of the occurrence rates and color are provided in right corner of the figure.

The examination of homology between spacers and phage sequences provides valuable insights into the range of potential targets and the regulatory and defensive activities against invasive DNA processes. All identified full CRISPR-Cas systems within *L. rhamnosus* strains demonstrate the capability to target at least one lactobacilli phage. In Figure 3, it is evident that strains Lrh3 and Lrh11 (1 phage) and LR2 (2 phages) exhibited the lowest number of *Lactobacillus* phages targeted by spacers within the CRISPR-Cas systems subtype II-A. In contrast, strains AS, CE1, and AMC143 displayed the highest number of *Lactobacillus* phages targeted by spacers within CRISPR-Cas systems subtype II-A. Figure 3 highlights *Lactobacillus* phages CL1, CL2, JNU_P10, iLp1308, PLE3, iLp84, PL-1, J-1, Lrm1, BH1, MLC-A, and LJ as the most potential targets for the CRISPR-Cas systems of *L. rhamnosus* strains. Further analysis revealed that *Lactobacillus* phage Lrm1 was targeted by the CRISPR-Cas systems of the most diverse *L. rhamnosus* strains, with only seven strains lacking the ability to target this phage. The presence of a functional CRISPR-Cas system in *L. rhamnosus* can enhance its resistance to phage infections, especially in industrial applications like yogurt production, where phage contamination can disrupt fermentation processes (Nami et al., 2020, 2021; Panahi et al., 2022). Additionally, *L. rhamnosus* is commonly used as a probiotic, and safeguarding its viability in the human gut is paramount. CRISPR-Cas systems play a crucial role in protecting these beneficial bacteria from phage attacks in the gut (Guo et al., 2023).

### Phylogeny analysis based on conserved sequences

**Figure 4:**
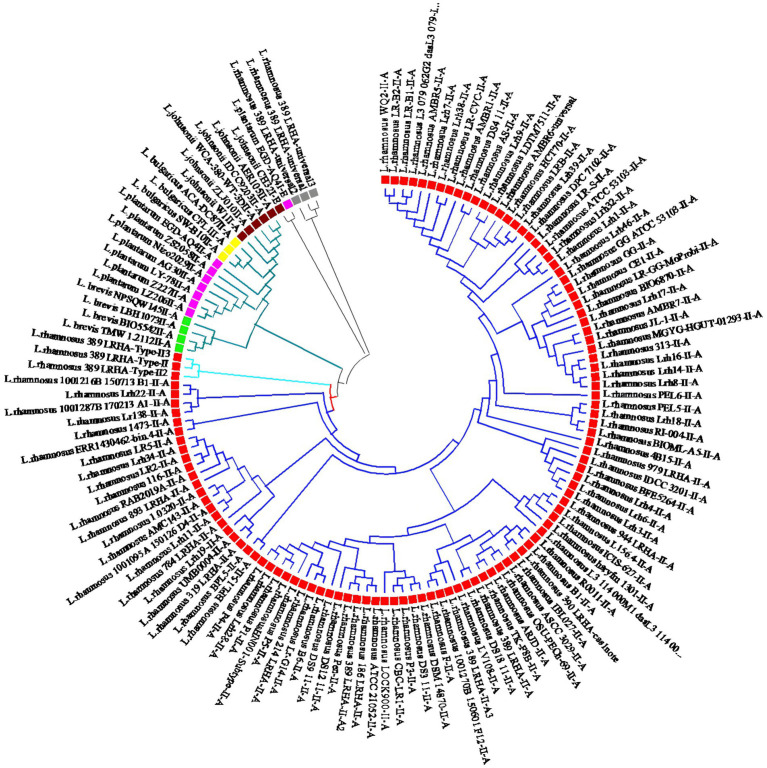
Phylogeny relationship between predicted CRISPR-Cas systems subtypes II-A in *L. rhamnosus* (Red square), *L. johnsonii* (Brown square), *L. plantarum* (Purple square), *L. brevis* (Green square), and *L. bulgaricus* (Yellow square). The phylogeny tree showed that the strains grouped in tow main clades. Clade one which consists of *L. rhamnosus* CRISPR-Cas systems further classified in two cluster I and II.

To explore the relationships among the identified CRISPR-Cas systems in *L. rhamnosus* and other *Lactobacillus* species like *Lactobacillus bulgaricus*, *Levilactobacillus brevis*, *Lactobacillus johnsonii*, and *Lactiplantibacillus plantarum*, multiple alignments and phylogenetic analyses based on Cas1 sequences were conducted. The phylogenetic investigation revealed that all CRISPR systems of subtype II-A can be categorized into two distinct clades. Notably, CRISPR-Cas systems subtype II-A in *L. rhamnosus* were classified under clade 1, while those in *L. bulgaricus*, *L. brevis*, *L. johnsonii*, and *L. plantarum* were classified under clade 2 (Figure 4). Within *L. rhamnosus*, the CRISPR-Cas systems subtype II-A were further categorized into two primary clusters, where cluster 1 consisted solely of strain 389_LRHA, and cluster 2 included all other strains. Moreover, major cluster 2 was subdivided into two sub-clusters. As depicted in Figure 4, strains 1001216B_150713_B1, Lrh22, 1001287B_170213_A1, and Lr138 were grouped in sub-cluster 1, while the remaining strains were grouped in sub-cluster 2.

In summary, this investigation suggests that while there is not significant diversity among the CRISPR-Cas systems identified in *L. rhamnosus* strains, substantial variation exists between CRISPR-Cas systems subtype II-A in *L. rhamnosus* and other lactobacilli. The diverse nature of these CRISPR-Cas systems underscores their inherent activity and importance in adaptive immunity.

## Data availability statement

The original contributions presented in the study are included in the article/Supplementary material, further inquiries can be directed to the corresponding author.

## Author contributions

YN: Formal analysis, Writing – review & editing. BP: Conceptualization, Formal analysis, Writing – review & editing. BD: Formal analysis, Writing – original draft.

## Footnotes

^1^ https://crisprcas.i2bc.paris-saclay.fr/
