# Supplementary material for: CRISPR-Cas systems feature and targeting phages diversity in Lacticaseibacillus rhamnosus strains
Source: Front Microbiol. 2023 Dec 6;14:1281307. doi: 10.3389/fmicb.2023.1281307 (PMC10731254; doi:10.3389/fmicb.2023.1281307)

**Supplementary figure 1.** Structural vartiation of identified CRISPR-Cas subtype II-A in *L.rhamnosus*


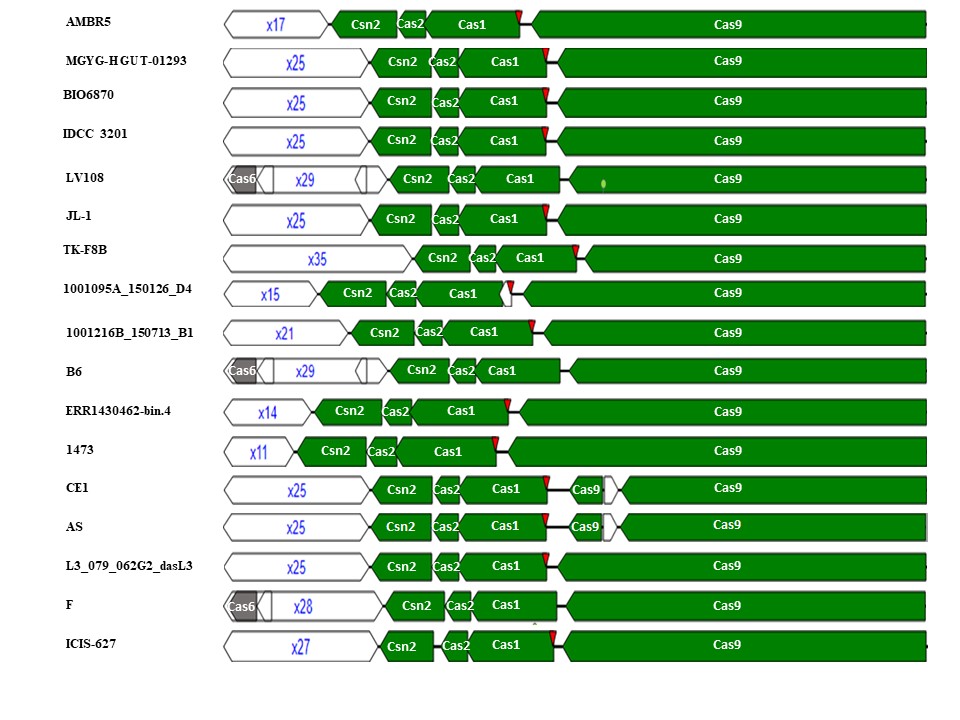

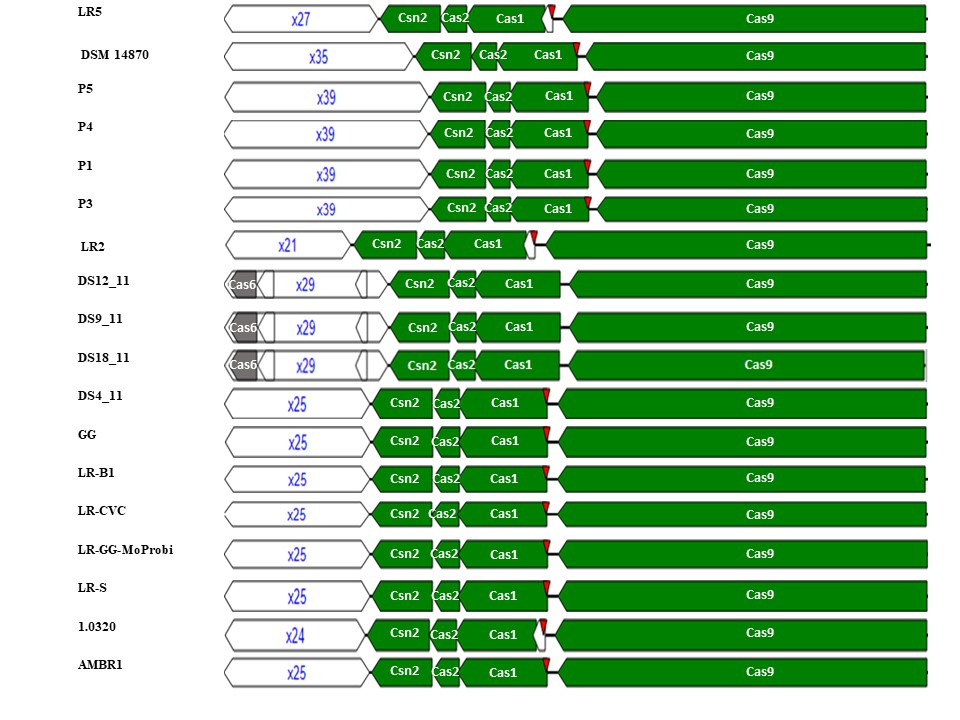

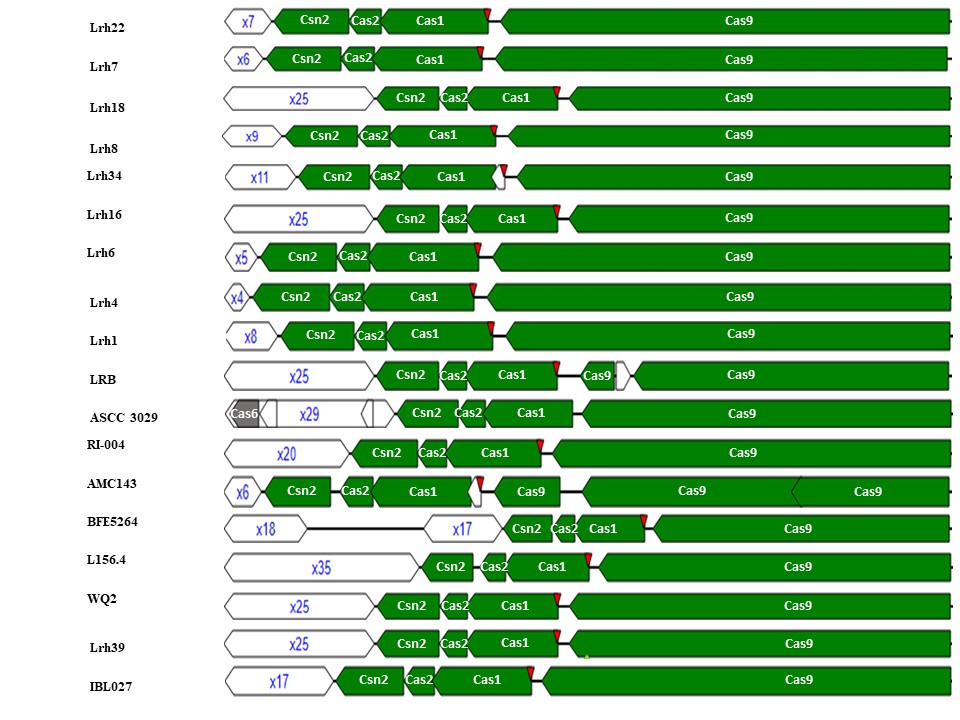

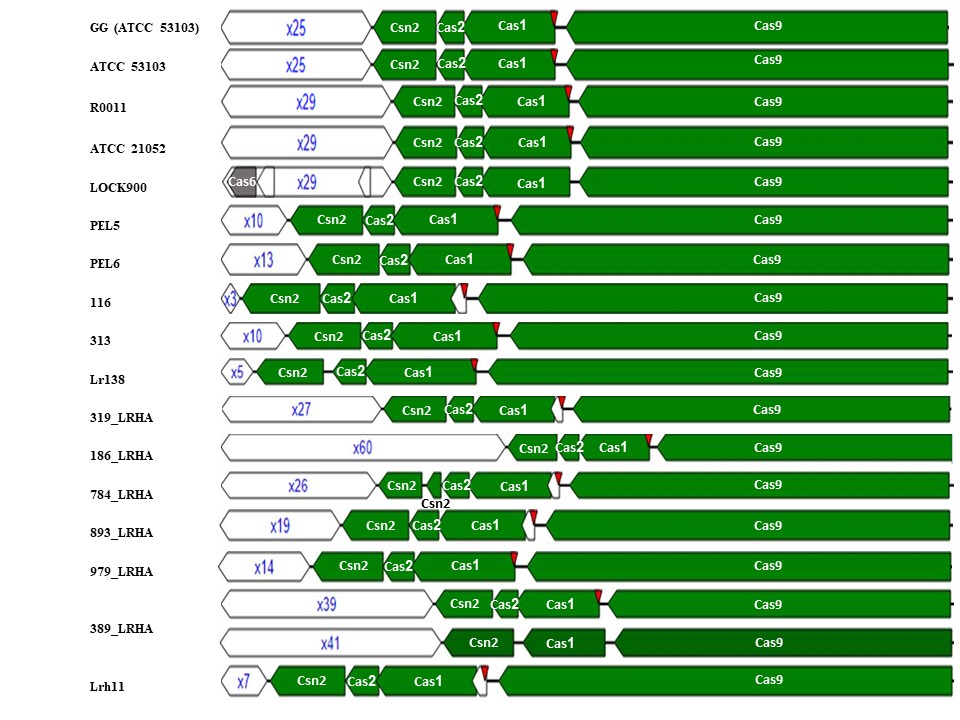

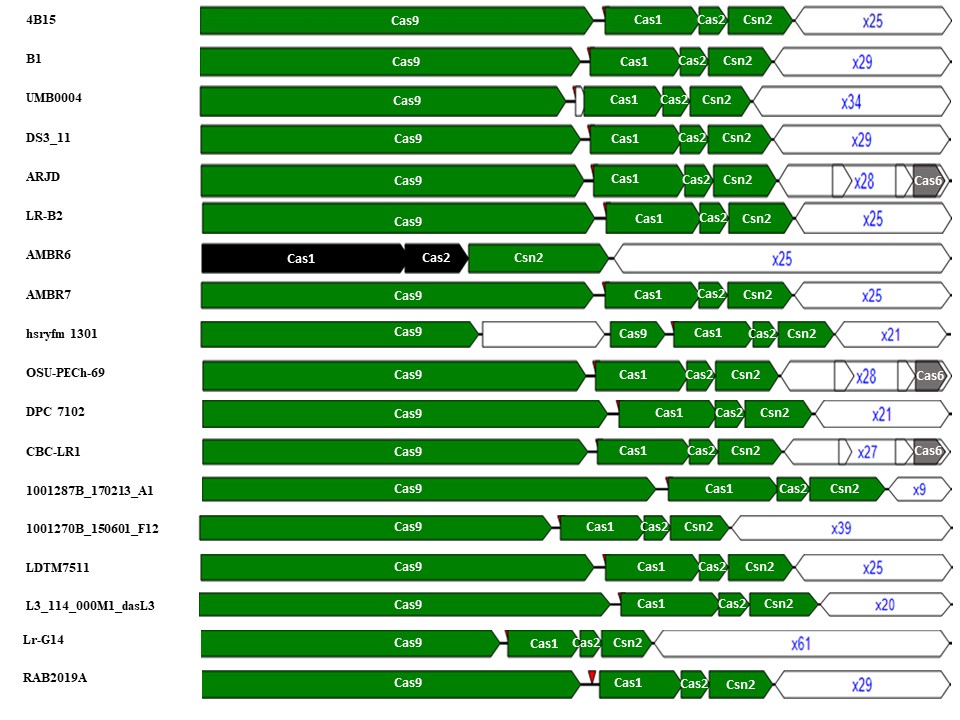

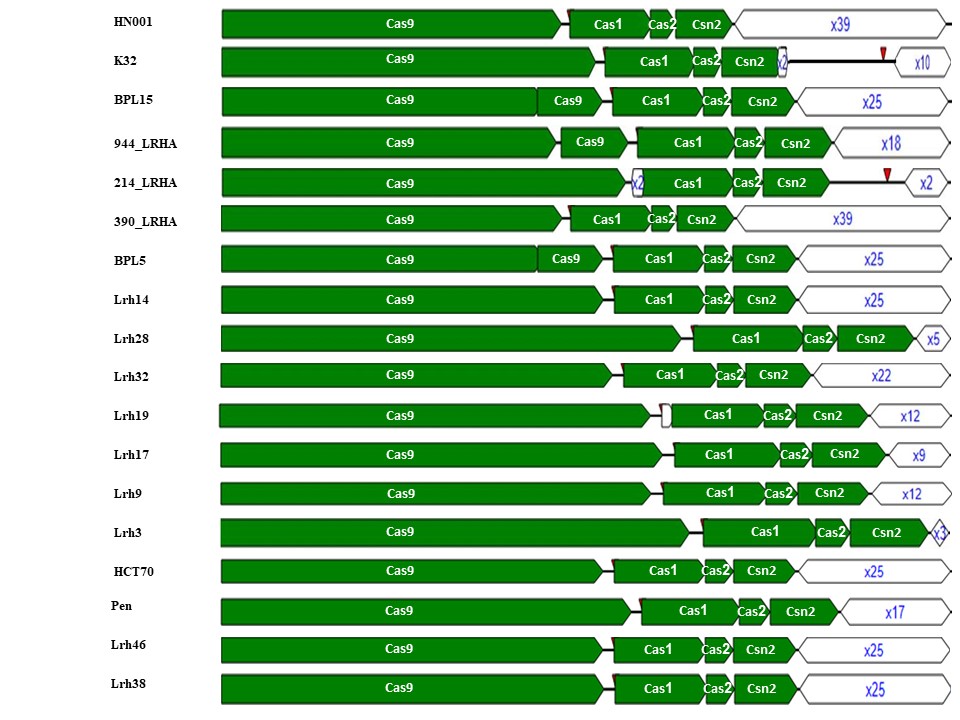

Supplement: Supplementary file 1 [file Table_1.DOCX]
