# Supplementary material for: CRISPR-Cas systems feature and targeting phages diversity in Lacticaseibacillus rhamnosus strains
Source: Front Microbiol. 2023 Dec 6;14:1281307. doi: 10.3389/fmicb.2023.1281307 (PMC10731254; doi:10.3389/fmicb.2023.1281307)
Supplement: Supplementary file 2 [file Table_2.DOCX]

Supplementary Table 1: Basic information of *Lacticaseibacillus rhamnosus* strains with a complete CRISPR-Cas system

| **Strains** | **Gen bank** | **Assembly name** | **Genome size** | **Sequencing level** | **Sequencing Date** | **Sequencing platform** |
| --- | --- | --- | --- | --- | --- | --- |
| 1.032 | GCF_006151905.1 | ASM615190v1 | 2941522 | Complete Genome | 6/9/2019 | PacBio; Illumina MiSeq |
| 116 | GCF_000801045.1 | ASM80104v1 | 2966235 | Contig | 12/11/2014 | 454 |
| 1473 | GCF_016887825.1 | ASM1688782v1 | 3082420 | Contig | 2/13/2021 | Illumina MiSeq |
| 1001095A_150126_D4 | GCF_015549225.1 | ASM1554922v1 | 2860739 | Contig | 11/17/2020 | Illumina HiSeq |
| 1001216B_150713_B1 | GCF_015557825.1 | ASM1555782v1 | 2903257 | Scaffold | 11/17/2020 | Illumina HiSeq |
| 1001270B_150601_F12 | GCF_015668455.1 | ASM1566845v1 | 2889915 | Scaffold | 11/23/2020 | Illumina HiSeq |
| 1001287B_170213_A1 | GCF_015558325.1 | ASM1555832v1 | 2815512 | Scaffold | 11/17/2020 | Illumina HiSeq |
| ERR1430462-bin.4 | GCA_905194935.1 | ERR1430462-mag-bin.4 | 2911850 | Contig | 2/4/2021 | Illumina HiSeq |
| GG (ATCC 53103) | GCF_000026505.1 | ASM2650v1 | 3010111 | Complete Genome | 9/2/2009 | Illumina HiSeq |
| hsryfm 1301 | GCF_008727835.1 | ASM872783v1 | 3067813 | Complete Genome | 9/29/2019 | Illumina MiSeq |
| L3_079_062G2_dasL3 | GCA_018368015.1 | ASM1836801v1 | 2651796 | Contig | 5/16/2021 | Illumina NovaSeq 6000 |
| L3_114_000M1_dasL3 | GCA_018364475.1 | ASM1836447v1 | 2846753 | Contig | 5/16/2021 | Illumina NovaSeq 6000 |
| LR-GG-MoProbi | GCF_004125465.1 | ASM412546v1 | 2844490 | Contig | 1/31/2019 | Illumina HiSeq |
| MGYG-HGUT-01293 | GCF_902381635.1 | UHGG_MGYG-HGUT-01293 | 3010111 | Complete Genome | 8/10/2019 | Illumina HiSeq |
| OSU-PECh-69 | GCF_013342125.1 | ASM1334212v1 | 3057669 | Contig | 6/14/2020 | Illumina NovaSeq |
| 186_LRHA | GCF_001062885.1 | ASM106288v1 | 3045143 | Scaffold | 7/10/2015 | Illumina HiSeq |
| 214_LRHA | GCF_001062955.1 | ASM106295v1 | 2962195 | Scaffold | 7/10/2015 | Illumina HiSeq |
| 319_LRHA | GCF_001064515.1 | ASM106451v1 | 2899444 | Contig | 7/10/2015 | Illumina HiSeq |
| 389_LRHA | GCA_001063295.1 | ASM106329v1 | 5899604 | Contig | 7/10/2015 | Illumina HiSeq |
| 390_LRHA | GCF_001064785.1 | ASM106478v1 | 2966799 | Scaffold | 7/10/2015 | Illumina HiSeq |
| 4B15 | GCF_002158925.1 | ASM215892v1 | 3047840 | Complete Genome | 5/30/2017 | PacBio |
| 784_LRHA | GCF_001067335.1 | ASM106733v1 | 2978131 | Scaffold | 7/10/2015 | Illumina HiSeq |
| 893_LRHA | GCF_001067625.1 | ASM106762v1 | 2960633 | Contig | 7/10/2015 | Illumina HiSeq |
| 944_LRHA | GCF_001068215.1 | ASM106821v1 | 2822292 | Scaffold | 7/10/2015 | Illumina HiSeq |
| 979_LRHA | GCF_001068015.1 | ASM106801v1 | 3014883 | Scaffold | 7/10/2015 | Illumina HiSeq |
| AMBR1 | GCF_901830405.1 | AMBR1 | 2969912 | Scaffold | 7/8/2019 | Illumina HiSeq |
| AMBR5 | GCF_901830425.1 | AMBR5 | 3027945 | Scaffold | 7/8/2019 | Illumina HiSeq |
| AMBR6 | GCF_901830355.1 | AMBR6 | 2992028 | Scaffold | 7/8/2019 | Illumina HiSeq |
| AMBR7 | GCF_901830365.1 | AMBR7 | 2998283 | Contig | 7/8/2019 | Illumina HiSeq |
| AMC143 | GCF_001982425.1 | ASM198242v1 | 2872742 | Contig | 2/1/2017 | 454; IonTorrent |
| ARJD | GCF_003573615.1 | ASM357361v1 | 2890298 | Contig | 9/18/2018 | Illumina HiSeq |
| AS | GCF_018286375.1 | ASM1828637v1 | 2935123 | Complete Genome | 5/6/2021 | Illumina MiSeq |
| ASCC 3029 | GCF_001831235.1 | ASM183123v1 | 2894885 | Scaffold | 10/21/2016 | Illumina HiSeq |
| ATCC 21052 | GCF_000235865.1 | ASM23586v1 | 2877033 | Scaffold | 11/18/2011 | Illumina |
| ATCC 53103 | GCF_000011045.1 | ASM1104v1 | 3005051 | Complete Genome | 9/25/2009 | Illumina HiSeq |
| B1 | GCF_002406705.1 | ASM240670v1 | 2908459 | Contig | 10/1/2017 | Illumina MiSeq |
| B6 | GCF_016599675.1 | ASM1659967v2 | 2924491 | Complete Genome | 1/11/2021 | PacBio Sequel |
| BFE5264 | GCF_001988935.1 | ASM198893v1 | 3114755 | Complete Genome | 2/6/2017 | PacBio |
| BIO6870 | GCF_008831425.1 | ASM883142v1 | 3006715 | Complete Genome | 10/7/2019 | Illumina Miseq |
| BPL15 | GCF_001368735.1 | CECT8361 | 3018284 | Scaffold | 4/20/2015 | Illumina Miseq |
| BPL5 | GCF_900070175.1 | CECT 8800 | 3024027 | Complete Genome | 3/1/2016 | Illumina Miseq |
| CBC-LR1 | GCF_014212185.1 | ASM1421218v1 | 2892155 | Scaffold | 8/16/2020 | Illumina MiSeq |
| CE1 | GCF_018141205.1 | ASM1814120v1 | 2935174 | Complete Genome | 4/27/2021 | Illumina MiSeq |
| DPC 7102 | GCF_014155845.1 | ASM1415584v1 | 3076900 | Contig | 8/11/2020 | Illumina HiSeq |
| DS12_11 | GCF_003061665.1 | ASM306166v1 | 2894311 | Contig | 4/14/2018 | Illumina MiSeq |
| DS18_11 | GCF_003052925.1 | ASM305292v1 | 2897248 | Contig | 4/17/2018 | Illumina MiSeq |
| DS3_11 | GCF_003052985.1 | ASM305298v1 | 2897523 | Contig | 4/17/2018 | Illumina MiSeq |
| DS4_11 | GCF_003052965.1 | ASM305296v1 | 2945494 | Contig | 4/17/2018 | Illumina MiSeq |
| DS9_11 | GCF_003061625.1 | ASM306162v1 | 2895476 | Contig | 4/14/2018 | Illumina MiSeq |
| DSM14870 | GCF_002287945.1 | ASM228794v1 | 3013149 | Complete Genome | 9/8/2017 | Illumina |
| F | GCF_018966895.1 | ASM1896689v1 | 2893669 | Contig | 6/20/2021 | IonTorrent |
| GG | GCF_003353455.1 | ASM335345v1 | 3010116 | Complete Genome | 8/2/2018 | Illumina HiSeq |
| HCT70 | GCF_001756565.1 | ASM175656v1 | 2975201 | Contig | 10/11/2016 | IonTorrent |
| HN001 | GCF_000173255.4 | ASM17325v2 | 2914408 | Scaffold | 9/12/2008 | Illumina HiSeq |
| IBL027 | GCF_002238035.1 | ASM223803v1 | 2898501 | Contig | 8/1/2017 | Illumina |
| ICIS-627 | GCF_021300595.1 | ASM2130059v1 | 3129120 | Contig | 12/27/2021 | Illumina MiSeq |
| IDCC3201 | GCF_009429065.1 | ASM942906v1 | 3064363 | Chromosome | 10/31/2019 | PacBio; Illumina MiSeq |
| JL-1 | GCF_015238575.1 | ASM1523857v1 | 3007502 | Complete Genome | 11/5/2020 | PacBio |
| K32 | GCF_000735255.1 | ASM73525v1 | 3016919 | Contig | 7/28/2014 | 454 |
| L156.4 | GCF_001991035.1 | ASM199103v1 | 2857730 | Contig | 2/9/2017 | Illumina MiSeq |
| LDTM7511 | GCF_017795605.1 | ASM1779560v1 | 3007472 | Complete Genome | 4/6/2021 | PacBio RSII |
| LOCK900 | GCF_000418475.1 | ASM41847v1 | 2883376 | Complete Genome | 7/8/2013 | Sanger dideoxy sequencing; 454; Illumina |
| Lr138 | GCF_001044075.1 | ASM104407v1 | 2737707 | Contig | 7/1/2015 | GS-FLX Titanium |
| LR2 | GCF_003046115.1 | ASM304611v1 | 2931951 | Scaffold | 4/9/2018 | Illumina MiSeq |
| LR5 | GCF_002286235.1 | ASM228623v1 | 2972590 | Complete Genome | 9/5/2017 | PacBio |
| LRB | GCF_001721925.1 | ASM172192v1 | 2934954 | Complete Genome | 9/8/2016 | PacBio |
| LR-B1 | GCF_004010975.1 | ASM401097v1 | 3007503 | Complete Genome | 1/10/2019 | PacBio |
| LR-B2 | GCF_004125395.1 | ASM412539v1 | 2942922 | Scaffold | 1/31/2019 | Illumina HiSeq |
| LR-CVC | GCF_004125455.1 | ASM412545v1 | 2941394 | Scaffold | 1/31/2019 | Illumina HiSeq |
| Lr-G14 | GCF_018449495.1 | ASM1844949v1 | 2920040 | Contig | 5/23/2021 | Illumina HiSeq |
| Lrh1 | GCF_001656755.1 | ASM165675v1 | 2789800 | Contig | 6/3/2016 | 454 |
| Lrh11 | GCF_001656735.1 | ASM165673v1 | 2897692 | Contig | 6/3/2016 | 454 |
| Lrh14 | GCF_001657115.1 | ASM165711v1 | 2899890 | Contig | 6/3/2016 | 454 |
| Lrh16 | GCF_001657085.1 | ASM165708v1 | 2925729 | Contig | 6/3/2016 | 454 |
| Lrh17 | GCF_001657075.1 | ASM165707v1 | 2921984 | Contig | 6/3/2016 | 454 |
| Lrh18 | GCF_001657055.1 | ASM165705v1 | 2934472 | Contig | 6/3/2016 | 454 |
| Lrh19 | GCF_001656685.1 | ASM165668v1 | 2937715 | Contig | 6/3/2016 | 454 |
| Lrh22 | GCF_001656655.1 | ASM165665v1 | 2966792 | Contig | 6/3/2016 | 454 |
| Lrh28 | GCF_001656925.1 | ASM165692v1 | 3004942 | Contig | 6/3/2016 | 454 |
| Lrh3 | GCF_001656915.1 | ASM165691v1 | 2824861 | Contig | 6/3/2016 | 454 |
| Lrh32 | GCF_001656545.1 | ASM165654v1 | 2933901 | Contig | 6/3/2016 | 454 |
| Lrh34 | GCF_001656765.1 | ASM165676v1 | 3007823 | Contig | 6/3/2016 | 454 |
| Lrh38 | GCF_002103155.1 | ASM210315v1 | 2878847 | Contig | 4/21/2017 | Illumina MiSeq |
| Lrh39 | GCF_002103145.1 | ASM210314v1 | 3004146 | Scaffold | 4/21/2017 | Illumina MiSeq |
| Lrh4 | GCF_001656875.1 | ASM165687v1 | 2830588 | Contig | 6/3/2016 | 454 |
| Lrh46 | GCF_002103215.1 | ASM210321v1 | 3088359 | Scaffold | 4/21/2017 | Illumina MiSeq |
| Lrh6 | GCF_001656835.1 | ASM165683v1 | 2853519 | Contig | 6/3/2016 | 454 |
| Lrh7 | GCF_001656815.1 | ASM165681v1 | 2866995 | Contig | 6/3/2016 | 454 |
| Lrh8 | GCF_001656535.1 | ASM165653v1 | 2866028 | Contig | 6/3/2016 | 454 |
| Lrh9 | GCF_001656785.1 | ASM165678v1 | 2885645 | Contig | 6/3/2016 | 454 |
| LR-S | GCF_004125475.1 | ASM412547v1 | 2927404 | Scaffold | 1/31/2019 | Illumina HiSeq |
| LV108 | GCF_013167115.1 | ASM1316711v1 | 2923663 | Complete Genome | 5/26/2020 | Illumina |
| P1 | GCF_002406795.1 | ASM240679v1 | 2967632 | Contig | 10/1/2017 | Illumina MiSeq |
| P3 | GCA_002406785.1 | ASM240678v1 | 2971889 | Contig | 10/1/2017 | Illumina MiSeq |
| P4 | GCF_002406745.1 | ASM240674v1 | 2950104 | Contig | 10/1/2017 | Illumina MiSeq |
| P5 | GCF_002406715.1 | ASM240671v1 | 2982246 | Contig | 10/1/2017 | Illumina MiSeq |
| PEL5 | GCF_000712505.1 | PEL5 | 2897032 | Contig | 6/26/2014 | 454 |
| PEL6 | GCF_000712515.1 | PEL6 | 2876865 | Contig | 6/26/2014 | 454 |
| PEN | GCF_002076955.1 | ASM207695v1 | 2884966 | Complete Genome | 4/3/2017 | Illumina HiSeq; Illumina MiSeq |
| R0011 | GCF_000235785.1 | ASM23578v2 | 2900620 | Contig | 11/18/2011 | 454 GS FLX Titanium |
| RAB2019A | GCF_018458775.1 | ASM1845877v1 | 2918787 | Scaffold | 5/24/2021 | Illumina HiSeq |
| RI-004 | GCF_001981725.1 | ASM198172v1 | 2915846 | Scaffold | 2/1/2017 | Illumina HiSeq |
| TK-F8B | GCF_015377485.1 | ASM1537748v1 | 3058443 | Complete Genome | 11/12/2020 | Oxford Nanopore GridION; Illumina HiSeq |
| UMB0004 | GCF_002848015.1 | ASM284801v1 | 2850032 | Scaffold | 12/28/2017 | Illumina HiSeq |
| WQ2 | GCF_002025085.1 | ASM202508v1 | 2960737 | Chromosome | 3/13/2017 | Illumina |
